# Microfluidic isoform sequencing shows widespread splicing coordination in the human transcriptome

**DOI:** 10.1101/gr.230516.117

**Published:** 2018-02

**Authors:** Hagen Tilgner, Fereshteh Jahanbani, Ishaan Gupta, Paul Collier, Eric Wei, Morten Rasmussen, Michael Snyder

**Affiliations:** 1 Brain and Mind Research Institute, Weill Cornell Medicine, New York, New York 10021, USA;; 2 Department of Genetics, Stanford University, Stanford, California 94304, USA;; 3 Arc Bio LLC, Menlo Park, California 94025, USA

## Abstract

Understanding transcriptome complexity is crucial for understanding human biology and disease. Technologies such as Synthetic long-read RNA sequencing (SLR-RNA-seq) delivered 5 million isoforms and allowed assessing splicing coordination. Pacific Biosciences and Oxford Nanopore increase throughput also but require high input amounts or amplification. Our new droplet-based method, sparse isoform sequencing (spISO-seq), sequences 100k–200k partitions of 10–200 molecules at a time, enabling analysis of 10–100 million RNA molecules. SpISO-seq requires less than 1 ng of input cDNA, limiting or removing the need for prior amplification with its associated biases. Adjusting the number of reads devoted to each molecule reduces sequencing lanes and cost, with little loss in detection power. The increased number of molecules expands our understanding of isoform complexity. In addition to confirming our previously published cases of splicing coordination (e.g., *BIN1*), the greater depth reveals many new cases, such as *MAPT*. Coordination of internal exons is found to be extensive among protein coding genes: 23.5%–59.3% (95% confidence interval) of highly expressed genes with distant alternative exons exhibit coordination, showcasing the need for long-read transcriptomics. However, coordination is less frequent for noncoding sequences, suggesting a larger role of splicing coordination in shaping proteins. Groups of genes with coordination are involved in protein–protein interactions with each other, raising the possibility that coordination facilitates complex formation and/or function. We also find new splicing coordination types, involving initial and terminal exons. Our results provide a more comprehensive understanding of the human transcriptome and a general, cost-effective method to analyze it.

RNA molecules carry information from the genome to produce the cell's functional machines, proteins, or act directly, for example, as long-noncoding RNAs. The complexity of the expressed transcriptome occurs, in part as a function of variability in transcription start sites (TSSs), exon inclusion, poly(A)-sites, and RNA modifications. All of these have been monitored with great success independently (Harrow et al. 2006; Kodzius et al. 2006; Pan et al. 2008; Sandberg et al. 2008; Wang et al. 2008; Mayr and Bartel 2009; Saletore et al. 2012; Batut et al. 2013). Transcript reconstruction with short reads is, however, difficult (Steijger et al. 2013; Tilgner et al. 2013), although there have been important advances (Behr et al. 2013).

A single gene can contain multiple alternative processing events, and such exon pairs can be separated by constitutive areas of the RNA molecule (MacLeod et al. 1985; Helfman et al. 1986; Fededa et al. 2005). If two alternative RNA processing events in the same gene are independent of one another, the probability of observing both in a molecule is simply the product of the probabilities of observing each of them. If this is not the case, we consider the two alternative RNA processing events to be coordinated. In this latter case, short-read sequencing does not inform on the status of both events within a single molecule—but isoform sequencing does. Nonrandom combination patterns of an internal exon and a 3′ exon were observed in the tropomyosin 1 gene (*TPM1*) (Helfman et al. 1986), and nonrandom combinations of alternative exons have been described in the fibronectin gene (Fededa et al. 2005). Recent targeted work has established the connectivity of alternative exons in four *Drosophila* genes (Bolisetty et al. 2015), mouse *Fn1*, and *Drosophila Dscam* (Roy et al. 2015) as well as mammalian neurexins (Schreiner et al. 2014; Treutlein et al. 2014). In neurexins, distant alternative exons were mostly independent. Earlier genome-wide work showed correlated inclusion patterns across tissues (Fagnani et al. 2007), but distinct isoform arrangements can underlie this observation. Yet, despite very important insights (Helfman et al. 1986; Cramer et al. 1997; Fededa et al. 2005; Fagnani et al. 2007; Tilgner et al. 2015), the understanding of exonic variability in full-length molecules is far from complete, because of the experimental difficulties of monitoring multiple variable sites in a single long molecule. Long-read RNA sequencing allowed the interrogation of splice sites en masse along the molecule (Tilgner et al. 2014, 2015). We previously analyzed ∼400- to 700-bp reads (Tilgner et al. 2013), revealing many long intergenic noncoding RNA (lincRNA) isoforms, but rarely described full-length isoforms. We and others (Au et al. 2013; Sharon et al. 2013; Tilgner et al. 2014) were able to obtain full-length isoforms using Pacific Biosciences sequencing technology (PacBio) (Eid et al. 2009). This revealed a variety of previously unappreciated isoforms, thus expanding our understanding. However, more quantitative aspects, such as isoform quantification and splicing coordination, remained difficult to address due to the lack of molecules analyzed. To increase sequenced molecule numbers and reduce length biases, we introduced synthetic long-read sequencing (SLR-RNA-seq), providing ∼5 million reads of genome assembly quality averaging 1.9 kb in length. Using SLR-RNA-seq, we demonstrated coordination of distant splicing events in the human brain (Tilgner et al. 2015). Oxford Nanopore has also been applied to isoform sequencing (Oikonomopoulos et al. 2016), and combining different data types for comprehensive analysis is promising (Sahraeian et al. 2017). Nevertheless, presently it appears unlikely that these methods could provide a full-length description of 10–100 million cellular RNA molecules—which is common in current short-read RNA-seq experiments. Here, we use the 10x Genomics system (Zheng et al. 2016) to generate linked short reads that tile across each molecule for 17–25 million RNA molecules, and an extension to ∼100 million molecules is straightforward. We have used these long-read technologies to search genome-wide for distant but dependent inclusion events of internal exons (Tilgner et al. 2015). Here, we aimed at analyzing whether distant coordinated exon pairs primarily affect coding regions and whether a large fraction of the human transcriptome is affected by coordinated exon usage.

## Results

### Outline of spISO-seq procedure

**Figure 1.**
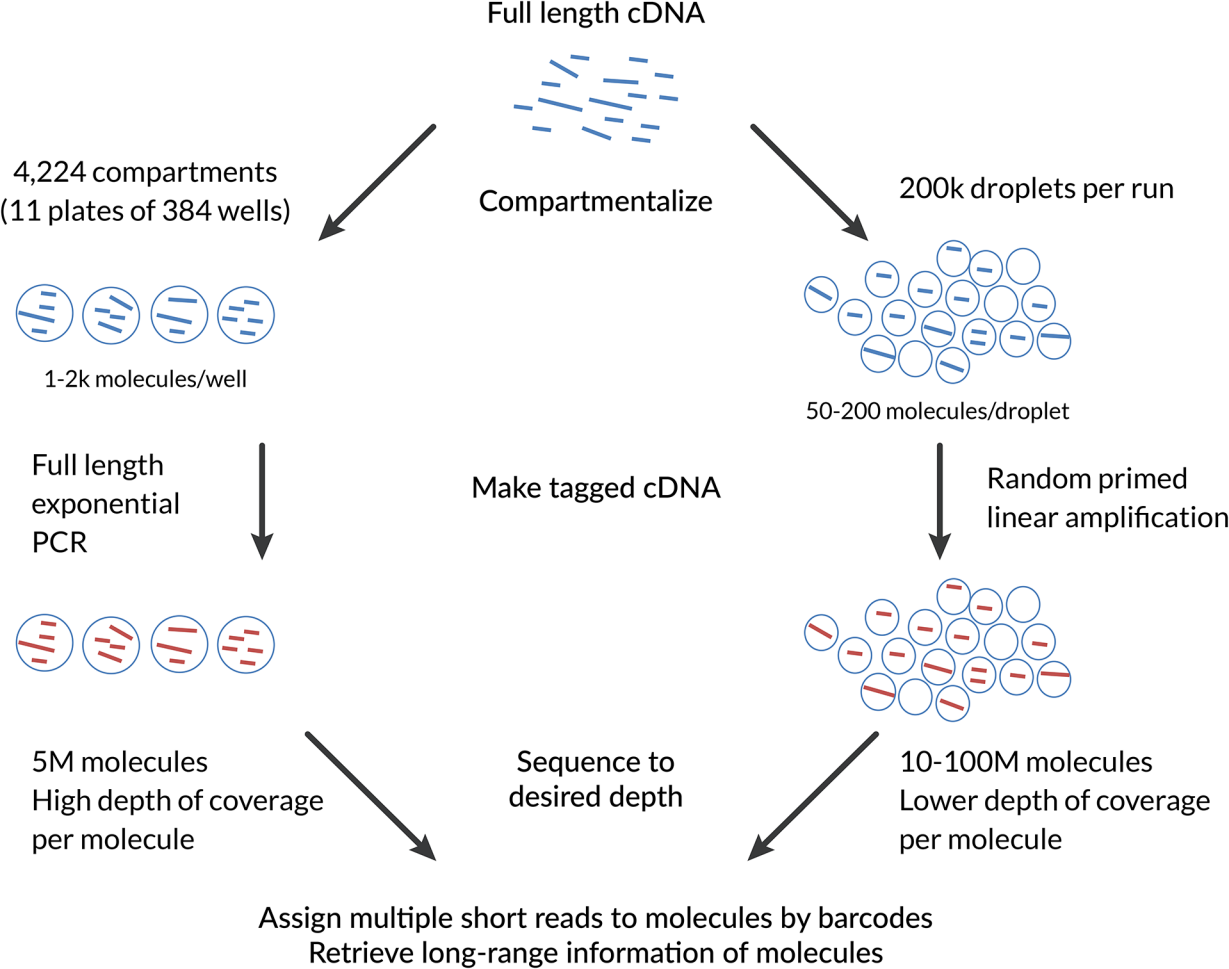
Comparative outline of the spISO-seq and the previously published SLR-RNA-seq approach. (1) Both approaches rely on the principle of compartmentalization. The fewer cDNA molecules are separated into one compartment, the lower the probability of having two nonidentical molecules from the same gene. SLR-RNA-seq employs 1000–2000 molecules per well on 384-well plates, while spISO-seq employs 50–200 molecules per droplet for a total of ∼200,000 droplets. (2) SLR-RNA-seq performs a full-length PCR that is exponentially amplifying all molecules in a well, while spISO-seq performs a linear randomly primed amplification. (3) In both approaches, the amplified product is short-read-sequenced using barcodes that identify the compartment (well or droplet of origin) and (based on that, most of the time only one molecule per gene is observed per compartment) the molecule of origin. (4) All short reads originating from the same molecule of origin are then collectively analyzed to retrieve long-range information within molecules.

The sparse isoform sequencing (spISO-seq) technology is currently implemented on the 10x Genomics GemCode platform (hereafter referred to as “GemCode”) but can, in principle, be implemented on other microfluidic devices. Droplet (or well)-based long-read sequencing relies on the statistical observation that a small sample of random cDNA molecules (10–1000 molecules) from a genome-wide experiment will contain, at most, one molecule for most genes. The spISO-seq approach exploits this by using the 200,000 droplets of the GemCode system to encapsulate 10–200 cDNA molecules in each droplet, while SLR-RNA-seq employed 384-well plates with 1000–2000 cDNA molecules per well. Both approaches amplify these molecules in a droplet (spISO-seq) or well (SLR-RNA-seq) separately and employ barcodes that assign the amplified molecules to the droplet or well of origin—and therefore to the original cDNA molecule with few exceptions. These barcodes are then observed in short-read RNA sequencing and serve to attribute tens to hundreds of short reads to one original RNA molecule. Advantages of spISO-seq with respect to SLR-RNA-seq include the higher number of total molecules, the reduced hands-on time during the experiment, and the smaller numbers of molecules per droplet. A clear disadvantage is the lower number of short reads for each original molecule, which renders direct genome-independent assembly of the total molecule difficult. We therefore aligned the spISO-seq short reads to the genome using STAR (Dobin et al. 2013) and analyzed sets of short reads from one droplet mapping to the same gene as a read cloud that describes the total isoform (Fig. 1). The overall spISO-seq logic can, in principle, be applied using microfluidic devices other than the 10x Genomics GemCode system we employed here. However, the exact parameters, such as input amount, may require adjustment on any non-GemCode system.

### Determining the concentration of input cDNA

**Figure 2.**
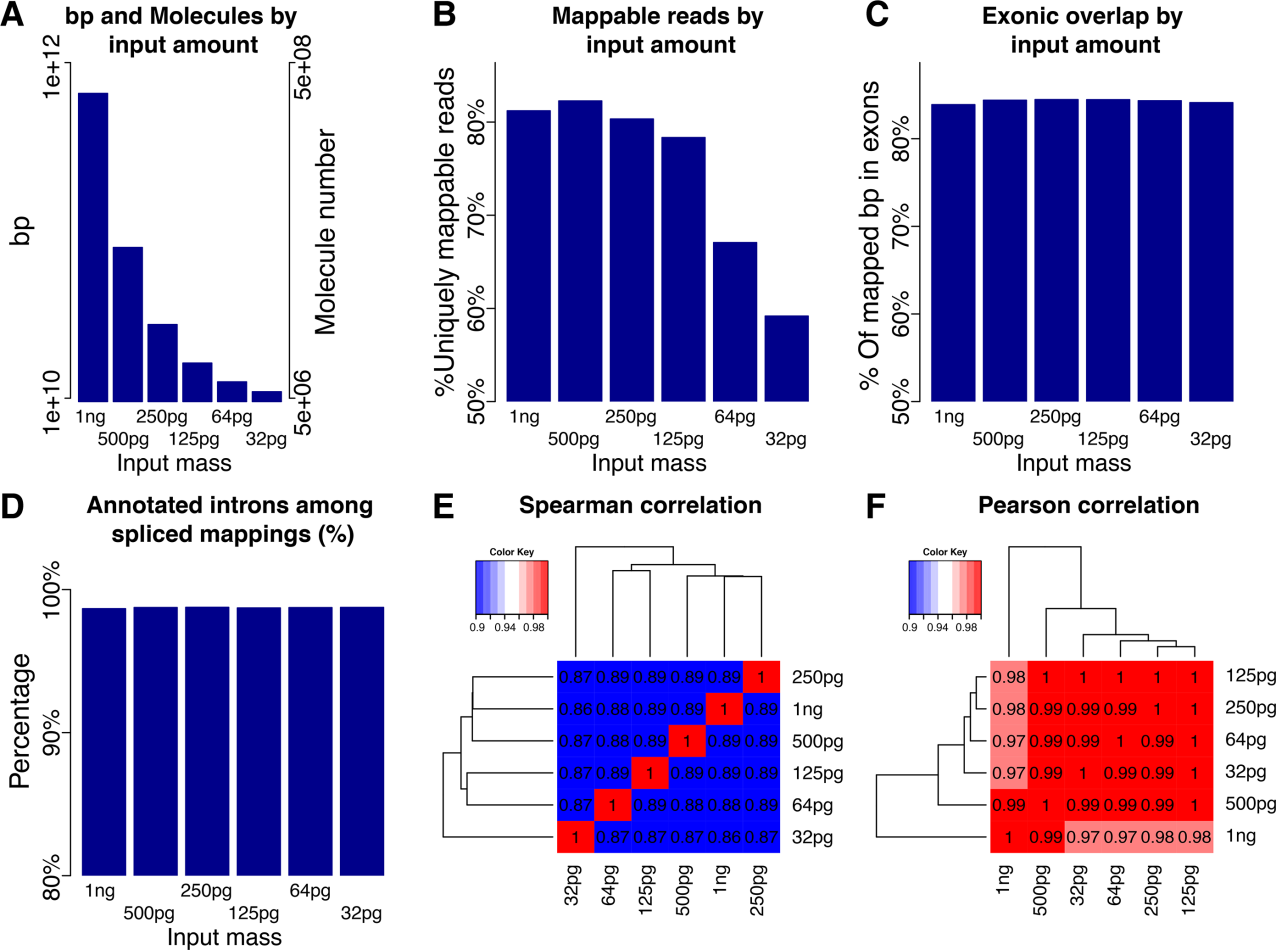
Exploration of low input capacities using shallow sequencing of a MiSeq. (*A*) Number of molecules and bases for different input amounts. (*B*) Percentage of reads that were uniquely mappable for all input amounts. (*C*) Percentage of mapped bases that fall onto annotated GENCODE exons for all input amounts. (*D*) Percentage of annotated introns among all spliced mappings for all input amounts. (*E*) Heat map of pairwise Spearman correlations of FPKMs for all input amounts. (*F*) Heat map of pairwise Pearson correlations of FPKMs for all input amounts.

We explored performing comprehensive transcriptome analysis using GemCode technology, which analyzes a small number of molecules separated into droplets. This technology performs multiple priming events on each single cDNA molecule, each containing the same barcode in a single droplet. One advantage of this technology is that it employs ∼1 ng of input cDNA, which corresponds to 10^12^ bp or 500 million molecules (assuming cDNA molecules of 2 kb). One Illumina lane of 200 million mappable paired-end 125-bp reads would give 0.05× coverage of these 10^12^ bp and PCR duplicates and unmappable reads would reduce coverage. We aimed at using lower input amounts to achieve higher coverage of molecules. To understand the behavior of the GemCode system, we employed 1 ng, and 500, 250, 125, 64, and 32 pg of dscDNA (Fig. 2A) from a previously used sample (Tilgner et al. 2015). We sequenced the resulting fragments using a MiSeq, observing fewer uniquely mappable reads for lower inputs. Approximately eighty percent of reads mapped uniquely for 125 pg or more, whereas for 64 and 32 pg, the percentages of reads that mapped uniquely was lower (Fig. 2B). We hypothesize that, with lower inputs, more primer-dimer-type molecules are created during linear amplification and that the resulting reads cannot be mapped to the genome. This differential behavior raises the issue as to whether the resulting mappings are genuine. Independently of input amount, we observed the same fraction of mapped base pairs in exonic regions (Fig. 2C), suggesting that uniquely mapped reads are genuine across input amounts. Likewise, the fraction of spliced read mappings that described annotated introns (GENCODE v24 annotation) (Harrow et al. 2006, 2012) was similar across inputs (Fig. 2D). The MiSeq data yielded fewer reads than there are molecules, and each molecule is thus mostly represented by one or zero read-pairs, just as in regular RNA-seq experiments.

We investigated if short-read gene expression measurements are consistent across input amounts using popular RNA-seq programs (Trapnell et al. 2010; Dobin et al. 2013) made for short-read RNA-seq experiments (Mortazavi et al. 2008; Nagalakshmi et al. 2008; Pan et al. 2008; Wang et al. 2008; Djebali et al. 2012). Correlation analysis showed that the 125-pg sample did not lead to disadvantages in quantification with respect to the other samples (Fig. 2E,F). For fragments per kilobase per million reads (FPKMs) of 2.31 to 4.62 (green region, Supplemental Fig. S1A), all inputs except the 32-pg were close to indistinguishable in a density plot, and for FPKMs of 4.62 or greater (white region, Supplemental Fig. S1A), we observed very high comparability. For FPKMs ≤ 2.31 (log(FPKM+1) ≤ 0.52) (gray/yellow region, Supplemental Fig. S1A), differences in the density plots were observable, presumably because low abundance genes are missed more frequently with lower inputs (Supplemental Fig. S1A). Heat map analysis tracks such disagreements to specific genes across all ranges of expression (Supplemental Fig. S1B). It is therefore possible to produce high quality RNA-seq data with inputs as low as 125 pg using the GemCode system.

### Gene detection using deep isoform sequencing

**Figure 3.**
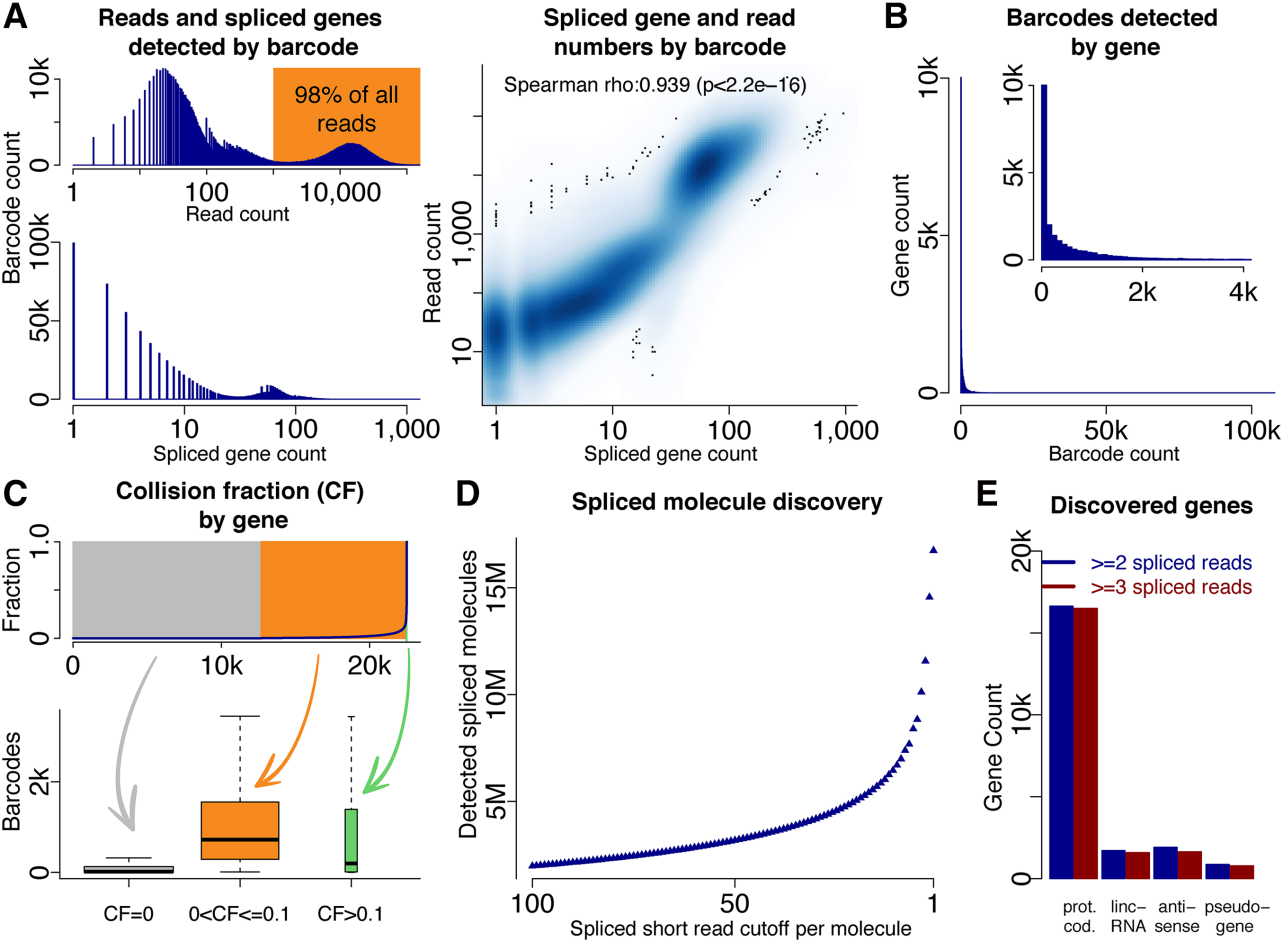
Molecule and gene identification using deep sequencing. (*A*) Histogram of read counts for all barcodes (*top left*), histogram of splice gene count for all barcodes (*bottom left*), and dotplot of spliced genes and short reads per barcode. (*B*) Histogram of barcodes per gene. (*C*) Percentage of barcodes with a collision for each gene; genes are ordered by collision fraction (*top*). Gene expression as measured by barcode number for many genes without collisions (gray), many genes with few collisions (yellow), and for very few genes with many collisions (green—not observable in *top* plot because of very low gene number). (*D*) Number of spliced molecules identified depending on how many spliced short reads identify an intron of the molecule's gene. (*E*) Numbers of genes identified in four gene classes (protein-coding genes, lincRNA genes, antisense genes, and pseudogenes).

We generated 2.01 billion paired-end 151-bp read-pairs (seven Illumina HiSeq lanes) from the 125-pg human brain dscDNA sample. We counted reads and intron-containing genes (referred to as “spliced genes” from here on) per barcode, with a spliced gene considered identified when one or more of its annotated introns was supported by a spliced junction read for this barcode. We detected a bimodal read count distribution per barcode, with most barcodes receiving 10–1000 reads and a population of barcodes receiving ∼10,000 reads. Barcodes with low (e.g., <1000) or very low (e.g., 1–100) read numbers are likely to be enriched in false barcode identifications. If droplets with 1000 (100 resp.) or less reads are considered false-positive barcode identifications, the barcode misidentification rate would be 1.8% (0.4% resp.) (Fig. 3A, left top). Most barcodes received 10–100 spliced genes but some barcodes noticeably fewer (Fig. 3A, left bottom). The latter barcode group may be erroneously identified barcodes. Barcodes with a higher number of spliced molecules also receive higher numbers of reads (Fig. 3A, right). The spISO-seq approach gives long-range isoform information for all genes in a droplet, for which only a single cDNA molecule is present in the droplet. When multiple isoforms of a gene are present in the same droplet, we refer to this situation as a “collision”—a situation that would lead to conflicting read mappings within a droplet. For example, if a given exon is both observed as included in a droplet but also as skipped, one possible explanation is a collision. However, a mismapped read giving a false-positive junction for a droplet can also lead to a false-positive collision. It is expected that, for highly expressed genes, it is more likely to have two molecules in a droplet (see Tilgner et al. 2015 for calculations). In our spISO-seq data, most spliced genes were detected in no more than 2000 (out of >200,000) droplets (Fig. 3B), making collisions unlikely. Nevertheless, we searched for conflicting spliced mappings within the same droplet (given by the barcode) to estimate collisions and calculated for each gene the fraction of barcodes with a collision (the “collision fraction”). For ∼20,000 spliced genes, this fraction is low (Fig. 3C). Genes with a slightly elevated collision fraction were observed with higher barcode numbers. This is consistent with more highly expressed genes having a higher collision probability (Tilgner et al. 2015). A few genes, however, had a very high collision fraction, even though they had lower expression (Fig. 3C), an observation that is not consistent with the theoretical collision calculations (Tilgner et al. 2015). A possible explanation for these cases is that false-positive mappings of some, but not all, reads in a droplet lead to false-positive conflicting splicing patterns within this droplet. Many barcodes (each representing a droplet) exhibited almost no collisions (including wrongly identified barcodes), and barcodes with collisions had more molecules (Supplemental Fig. S2A,B). To estimate the number of detected spliced molecules, we mapped short reads against the genome and splice junctions from the GENCODE annotation (Harrow et al. 2006; Derrien et al. 2012) and our previous SLR-RNA-seq data (Tilgner et al. 2015). Using multiple cutoffs (i.e., 1, 2, 3, …, 100), we considered a molecule of a gene identified for a given barcode when at least that many spliced reads of a barcode spanned an intron of the gene (Methods). With one or more spliced short reads, we detect ∼16.7 million spliced molecules, with two or more, 14.6 million, and with three or more, 11.6 million spliced molecules; 16.7 million match surprisingly well with theoretical expectations (Methods), although it likely includes some false-positive identifications (Methods; Fig. 3D). We previously estimated (Sharon et al. 2013) that about two-thirds of all molecules in long-read data sets are spliced. Adding unspliced molecules onto the above estimations, we arrive at 17.4 million (from 11.6 million spliced molecules) to 25 million (from 16.7 million spliced molecules) total molecules. Supplemental Table S1 shows, for each intron in the genome, the barcodes that span it and allows reconstruction of isoforms. Most identified genes were protein-coding genes, consistent with their higher average expression (Derrien et al. 2012). However, >1000 lncRNA-, antisense-RNA, and spliced pseudogenes were also detected, allowing better interrogation of lncRNAs, which remain of high interest (Mele et al. 2017). For pseudogenes, we advise caution, because gene identification is based on short-read mapping, which for pseudogenes is error-prone (Fig. 3E).

### Characteristics of detected genes

**Figure 4.**
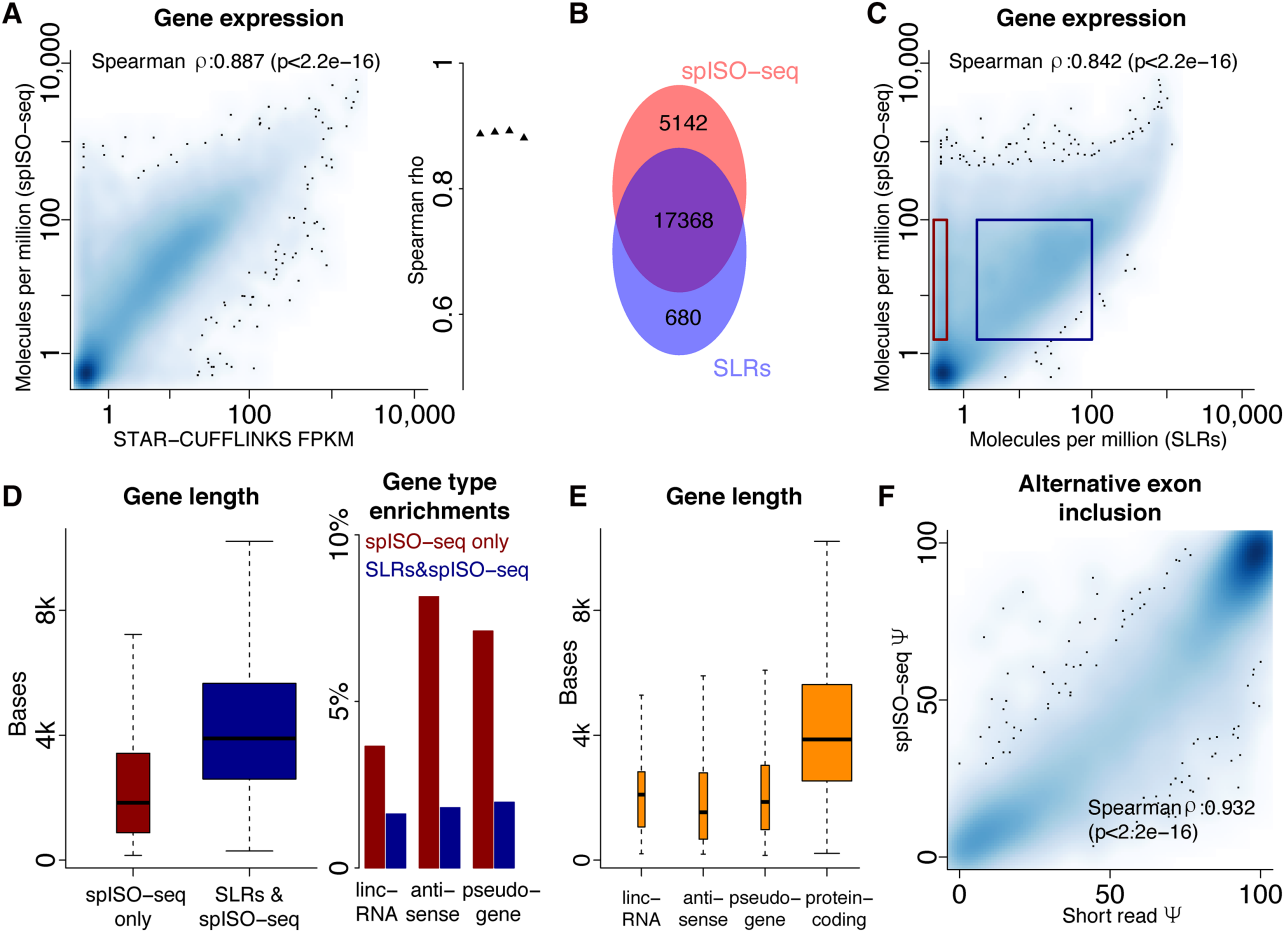
Gene quantification. (*A*) Dotplot of gene expression from published short-read data (Li et al. 2014) and molecules per million of spISO-seq. (*B*) Overlap of genes identified by spISO-seq's linked reads and SLR-RNA-seq's SLRs. (*C*) Dotplot of gene expression from published synthetic long-read data (Tilgner et al. 2015) and molecules per million of spISO-seq. (*D*) Gene length and gene type enrichments for genes found only with spISO-seq and those found with spISO-seq and SLR-RNA-seq (Tilgner et al. 2015). (*E*) Length for mature RNAs for four different gene classes. (*F*) Dotplot of Ψ-values of short-read RNA sequencing (*x*-axis) and of spISO-seq (*y*-axis).

We calculated gene expression values (molecules per million [MPM]) (Methods) for each gene in our spISO-seq data and compared them to FPKMs, deduced from regular short-read RNA sequencing (Li et al. 2014) using STAR (Dobin et al. 2013) and Cufflinks (Trapnell et al. 2010). We found high correlation (Fig. 4A, left) for all short-read data replicates (Fig. 4A, right). Note that we use gene expression values because they can be easily calculated from short reads; however, the real value of spISO-seq is on the isoform level. Comparing the genes detected by spISO-seq and those detected in the SLR-RNA-seq data (Tilgner et al. 2015) from the same sample, we found 17,368 spliced genes in both data sets; 680 genes detected only using SLR-RNA-seq and 5142 genes found only with spISO-seq (Fig. 4B). Importantly, spISO-seq expression and SLR-RNA-seq expression showed a strong Spearman correlation of 0.842 (Fig. 4C). Genes detected only by spISO-seq were shorter than those detected by both approaches, presumably due to a bias against very short molecules (Tilgner et al. 2015) in SLR-RNA-seq (Fig. 4D, left). Genes detected only by spISO-seq were also enriched in lncRNA, antisense, and pseudogenes (Fig. 4D, right). These two enrichments (shorter length and enrichment in lncRNA, antisense, and pseudogenes) appear linked because lncRNA, antisense, and pseudogenes as groups are shorter than protein-coding genes (Fig. 4E; Methods). Note that, for pseudogenes, SLR-RNA-seq identifications (Tilgner et al. 2015) are more trustworthy because they are not based on short-read mapping. Percent spliced-in values of alternative exons from short-read sequencing in human brain (Li et al. 2014) and in our spISO-seq were concordant with a Spearman correlation of 0.93 (Fig. 4F) and a Pearson correlation of 0.96.

### Coordination of alternative internal exons

In the GENCODE (version 24) annotation, 24.8% of protein-coding genes contain two pure exon-skipping events that are separated by constitutive exons. We define a “pure exon-skipping event” as an exon that is either included or skipped in all overlapping transcripts, without the interference of annotated alternative splice sites, poly(A)-sites, transcription start sites, or intron retention. However, 60.5% of protein-coding genes contain two nonconstitutive exons that are separated by one or more constitutive exons. The question of coordination of multiple distant alternative sites is therefore relevant for many genes. We recently revealed a limited number of exon pairs that are distant in mRNAs but show coordinated inclusion patterns in brain (Tilgner et al. 2015). Here, we improved the detection algorithm and devised a similar procedure for spISO-seq data (Methods). For splicing coordination, we use very stringent mapping parameters (Methods) and correct for multiple testing using the Benjamini-Yekutieli method (Benjamini and Yekutieli 2001). We found 125 genes with at least one coordinated pair at a false discovery rate (FDR) of 0.05 (Supplemental Fig. S3A; Supplemental Table S2) and 110 genes using the SLR-RNA-seq data (Methods; Supplemental Table S3). The neurexin genes with mostly independent alternative exon pairs (Schreiner et al. 2014; Treutlein et al. 2014) serve as a negative control: Indeed, despite testing 24 alternative exon pairs for dependence, none of these neurexin exon pairs was found to be coordinated. Removing spISO-seq barcodes with <1000 short reads (which contain false-positive molecule identifications), exon pairs of seven (*PPFIBP1*, *KIAA1217*, *RECK*, *PPA2*, *PPFIA2*, *TPM1*, and *CCDC25*) of the 125 genes showed increased corrected *P*-values (from <0.05 to 0.05–0.16). These are either cases of false-positive coordination events (due to false-positive molecule identification events) or real coordination events for which the “≥1000 reads” cutoff removed real molecules and increased *P*-values.

**Figure 5.**
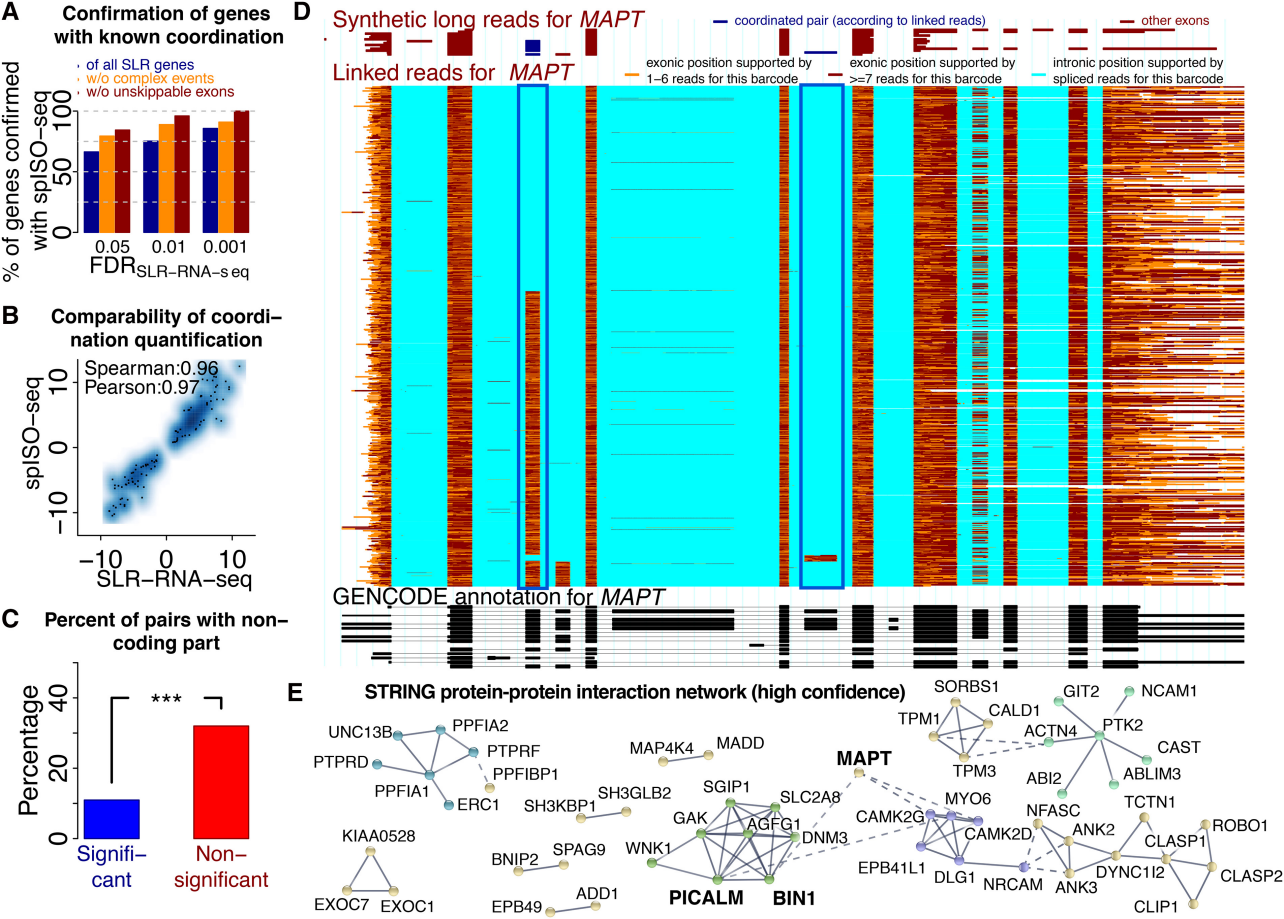
Coordinated exon pairs and influences on protein–protein interactions. (*A*) Percentage of genes with coordination events found by SLR-RNA-seq at three different FDRs that are also found with spISO-seq at FDR of 0.05. Blue bars: all SLR-RNA-seq coordination genes; orange bars: only SLR-RNA-seq genes, in which most molecules (Methods) show only exon inclusion and exon exclusion; brown bar: only SLR-RNA-seq genes, in which most molecules (Methods) show only exon inclusion and exon exclusion and where skipping events are mappable using short reads and STAR (Dobin et al. 2013). (*B*) Dotplot for extent of coordination according to SLR-RNA-seq and spISO-seq for cases in which both technologies indicate coordination. (*C*) Percentage of genes in which coordinated exons contain noncoding sequence for genes with coordination (FDR < 0.05) and without. (*D*) Single gene view for the *MAPT* gene, the center of all tauopathies. *Bottom*, black track: GENCODE annotation. *Middle*, colored track: spISO-seq data, with each line representing one molecule. *Top*, red-brown track: SLR-RNA-seq data with each line representing one molecule. Blue boxes highlight the inclusion of two alternative exons, whose inclusion is anticorrelated. (*E*) Protein–protein interaction network for genes with splicing coordination.

Sixty-six percent of the 110 genes with coordination (FDR = 0.05) according to SLR-RNA-seq were among the 125 genes with coordination using spISO-seq (FDR = 0.05). The confirmation rate gradually rose to 86% for SLR-RNA-seq FDRs of 0.01 and 0.001. Removing exon pairs with complex events (i.e., intron retention and/or alternative splice sites are observed in addition to the exon-inclusion and exon-skipping isoform) from the SLR-RNA-seq list led to higher confirmation rates. Finally, removing exon pairs with a junction that could only be mapped using SLRs, but not with short reads, increased the confirmation rate to 96% and 100% (FDRs = 0.01 and 0.001) (Fig. 5A). Presumably, nonunique junctions limit the ability of the linked read approach—or differences between GMAP (Wu and Watanabe 2005) and STAR (Dobin et al. 2013) cause the nonperfect overlap in the entire set. Overall, there is agreement between both technologies, although complex splicing events and differences in short- and long-read mappability introduce some disagreement. Differences in bioinformatic implementations that are tuned to take advantage of each method may also contribute to this discrepancy (Fig. 5A; Methods). Log-odds-ratios—a measure of coordination extent—correlated highly for exon pairs that were coordinated with both technologies (Spearman correlation of 0.96) (Fig. 5B), as well as for those, albeit less, that were only significant in the spISO-seq approach (Spearman correlation of 0.90) (Supplemental Fig. S3B). Pairs that were only coordinated in spISO-seq show higher molecule numbers in spISO-seq, illustrating statistical advantages with deeper sequencing (Supplemental Fig. S3C).

For genes with exactly one alternative exon pair that is entirely coding, in 67% (34 out of 51) of the cases both exons had lengths divisible by three, thus keeping the reading frame. In the remaining 33% of the cases, nonsense-mediated decay (NMD) may have contributed to the observation of coordination, by degrading RNA molecules carrying specific exon combinations. Coordinated exon pairs showed a relatively wide distribution of bases in their intermediate exons according to the GENCODE annotation—at least in comparison to customary Illumina sequencing fragment length. A considerable number of coordinated exon pairs are separated by more than 1000 bp on RNA molecules (Supplementary Fig. S3D). Only two coordinated exon pairs could be observed in four lanes of Illumina sequencing (Li et al. 2014) after PCR duplicate removal. However, deeper, 150-bp paired-end sequencing is expected to yield more coordinated exon pairs for a subset of distances covered by the fragment length.

Tested exon pairs that included noncoding sequence were coordinated less often than those that were entirely coding, suggesting that coordination acts predominantly on proteins (Fig. 5C). Of note, the majority of pairs with noncoding sequence come from protein-coding genes, because of lower lincRNA expression, in the literature (Derrien et al. 2012) and in our data set. To control for informative read number, we selected a distribution of purely coding exon pairs and a second distribution involving noncoding sequence with matched read numbers. We found the former to have twofold higher frequency of coordination (two-sided Fisher's exact test, *P* < 0.007) (Methods; Supplemental Fig. S3E). A gene our previous approach had not identified, and an example of coordination between coding exons, is *MAPT*, which forms the basis of all tauopathies (Rademakers et al. 2004; Wang and Mandelkow 2016). We now have a much larger number of identified molecules—especially for the second alternative exon (Fig. 5D). Among the many SLR-RNA-seq coordinated genes (Tilgner et al. 2015) that we can confirm are *EXOC7* (Supplemental Fig. S4) and *BIN1*,the second most Alzheimer's disease (AD)-associated gene (Supplemental Fig. S5; Lambert et al. 2013). In both genes, we observe rare but existing intron retention events, which have attracted considerable interest in recent times (Braunschweig et al. 2014; Jacob and Smith 2017). The increased coordination between entirely protein-coding exons prompted us to investigate the involved proteins. Genes with coordination appear to harbor clusters of known protein–protein interactions (Szklarczyk et al. 2017). One such cluster appears to be linked to AD (with *MAPT*, *BIN1*, *PICALM*). Another cluster contains a number of cell adhesion molecule genes (*NRCAM*, *NFASC*), and yet another cluster—a group of actin-cytoskeleton remodeling genes (*TPM1*, *TPM2*) (Fig. 5E). This suggests that, in addition to known influences of alternative splicing on protein–protein interactions (Yang et al. 2016), these protein–protein interactions may be governed by complex nonrandom exon-pairing.

### Effects of lower coverage of molecules

We assessed the impact of devoting less sequencing depth to the same number of molecules, by limiting our analyses to two of the seven lanes of Illumina data. This 71% decrease in sequencing depth led to 37.5% of molecules and 34% of splicing events not having coverage (Supplemental Fig. S6A). Some of these losses may represent false-positive identifications with few reads only. However, when it comes to the utility of this approach to detect coordination events, this 71% decrease in resources still recovered 99 genes with coordinated splicing—of the original 125 (Supplemental Fig. S6B). Quantitative coordination extent, measured by log-odds-ratios, correlated highly between the two-lane and the seven-lane approach (Supplemental Fig. S6C). In the case of *EXOC7*, we observed that most, but not all, positions of molecules were still supported, albeit by fewer reads (Supplemental Fig. S6D).

### Coordination of first alternative donors and last alternative acceptors

**Figure 6.**
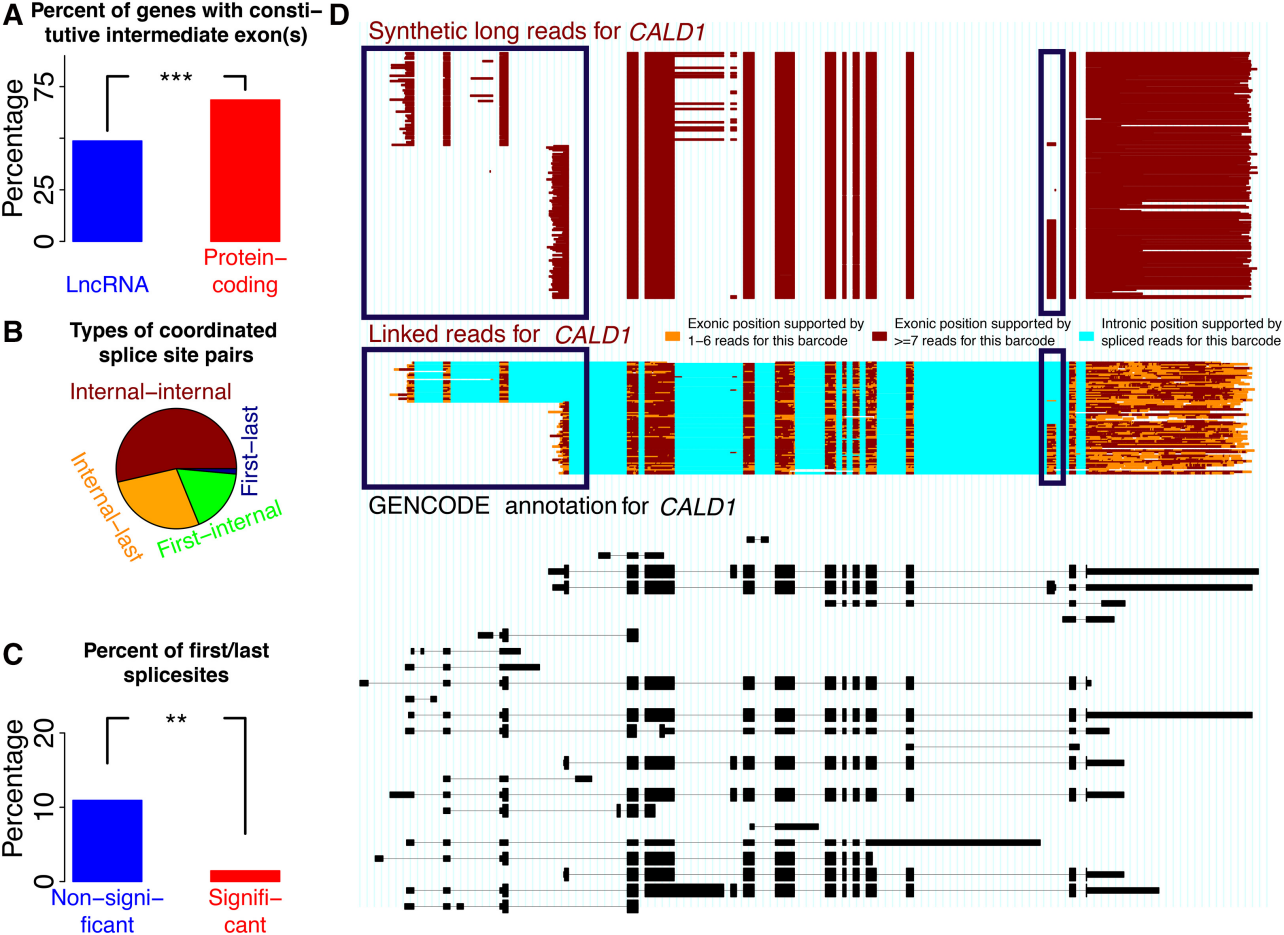
Coordination between first alternative donors and last alternative acceptors. (*A*) Percent of exon pairs that are always separated by at least one intermediate exon for lincRNAs and for protein-coding genes. (*B*) Frequency among all coordinated pairs of pairs of internal splice sites (“Internal-internal”), pairs of an internal and a last splice site (“Internal-last”), pairs of a first splice site and an internal exon (“First-internal”), and pairs of a first and a last splice site (“First-last”). (*C*) Percentage of pairs of a first and a last splice site among coordinated (FDR < 0.05) and noncoordinated pairs. (*D*) *Bottom*, black track: GENCODE annotation. *Middle*, colored track: spISO-seq data, with each line representing one molecule. *Top*, red-brown track: SLR-RNA-seq data with each line representing one molecule. Blue boxes highlight first exon and TSS choice (*left* blue boxes) and internal exon inclusion (*right* blue boxes). Inclusion of the alternative internal exon occurs only when the downstream first exon/TSS is chosen.

Our previous research (Tilgner et al. 2015) focused on internal exon coordination, in the absence of complex events (e.g., intron retention events, alternative acceptor and donors). Given spISO-seq advantages for long molecules, we focused on long-distance coordination. We considered genes that had at least one alternative donor that was located upstream in the gene of an alternative acceptor. We assessed the number of genes in which these two alternative splicing events were separated by one or more constitutive exons. For protein-coding genes, this was the case in 69% of all genes, and for lncRNAs, in about half of all cases (Fig. 6A). This reduced presence of constitutive exons in lncRNAs is consistent with increased isoform diversity through skipping of exons previously considered constitutive (Sharon et al. 2013; Tilgner et al. 2013, 2014, 2015) and universal alternative splicing (Deveson et al. 2017) in lincRNAs. This procedure performs one test per gene, and we correct for multiple testing using the Benjamini-Hochberg method (Benjamini and Hochberg 1995). Despite testing the most upstream alternative donor and the most downstream alternative acceptor, an absolute majority of significantly coordinated pairs were splice sites of internal exons. Only once did we observe a first donor being coordinated with a last acceptor, while internal splice sites were frequently paired to a first or last splice site (Fig. 6B). Pairs of first donors and last acceptors were thus underrepresented among the coordinated pairs (two-sided Fisher's exact test, *P* < 0.01) (Fig. 6C), while pairs of internal donors and last acceptors were slightly overrepresented (two-sided Fisher's exact test, *P* < 0.04). Given that last alternative acceptors can also occur as part of distant 3′ exons, this observation is consistent with previously observed coordination of 3′ exons and internal exons (Helfman et al. 1986) and our previous model: that alternative poly(A)-site choice can influence internal splice site selection by changing the rate of cotranscriptional splicing (Tilgner et al. 2012). Pairs of alternative first donors and internal acceptors, as well as pairs of two internal splice sites, showed no enrichment among the coordinated pairs. An alternative first donor linked to the choice of an internal exon is shown in Figure 6D. The two splice sites are separated by nine constitutive exons. First alternative donor choice is perfectly correlated with TSS choice, so that the driving force may in fact be TSS and promoter choice—this is consistent with work linking promoters and splicing patterns (Cramer et al. 1997; Fededa et al. 2005) and DNA loops between TSS and internal exons (Mercer et al. 2013; Curado et al. 2015). In this example, including the third-from-last exon is never observed when the gene is transcribed from the upstream TSS. We advise caution with such cases of alternative TSSs (and poly(A)-sites) because these can affect molecule length drastically, but cases (such as *CALD1*) observed with two technologies (i.e., spISO-seq and SLR-RNA-seq) are less likely to be artifacts.

### Estimation of coordination extent

**Figure 7.**
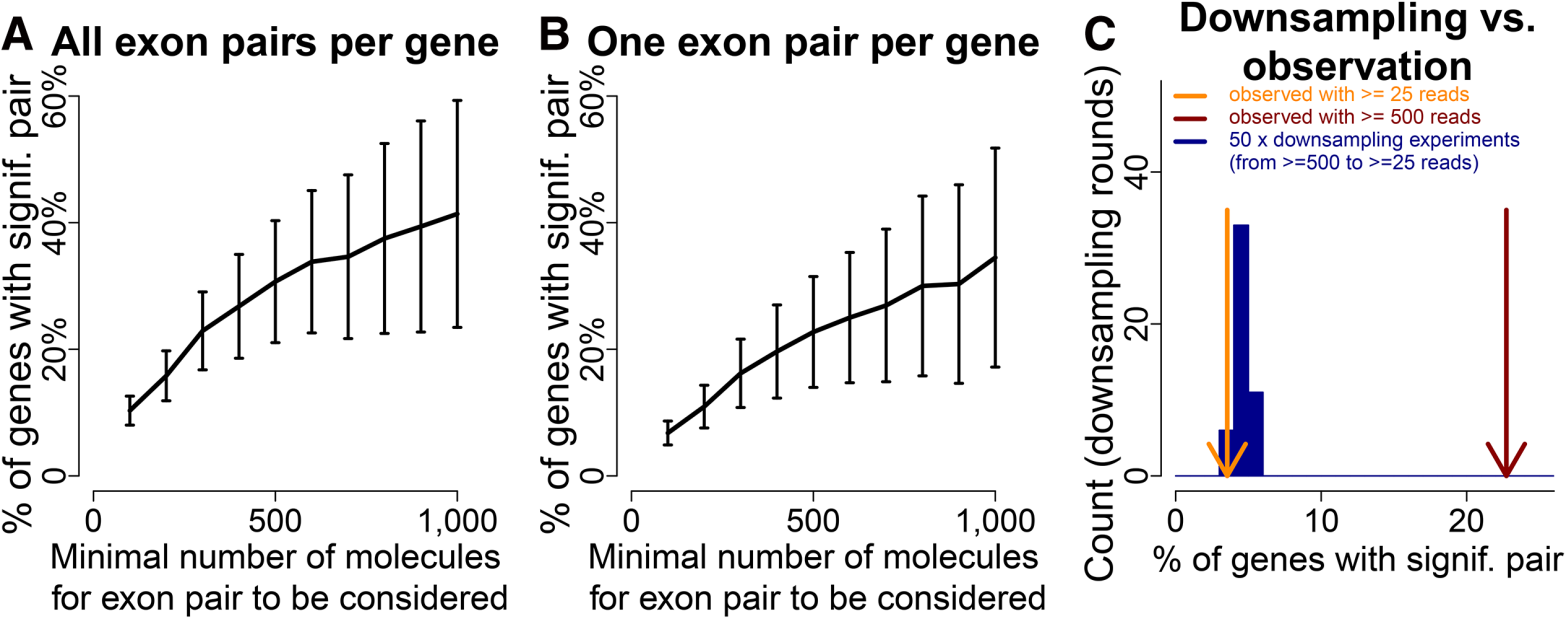
Estimation of genes with coordination genome-wide. (*A*) Percent of genes (among genes with one tested exon pair at a given cutoff) that show at least one coordinated exon pair with *P* < 5 × 10^−7^ and absolute value log-odds-ratio of 0.5 or above. Vertical bars indicate 95% confidence intervals. (*B*) Same figure as *A*, considering only one exon pair per gene: the one with the highest number of informative reads. Vertical bars indicate 95% confidence intervals. (*C*) Orange arrow indicates percentage of genes with ≥25 informative reads that have a coordination event. Red arrow indicates the same percentage for genes with 500 informative reads. Blue distribution shows 50 lists of exon pairs, down-sampled from the “≥500 informative read” data to the “≥25 informative read data.”

Increased sample size yields increased power to reveal statistical significance. Since for nonrandom exon pairing small read numbers (i.e., 0–100) for an exon pair limit detection in lowly expressed genes, we aimed at estimating the percentage of genes (that have multiple separate variable exons) with coordination. We considered, for multiple cutoffs, the exon pairs having at least this many reads. At a cutoff of 25, 5.6% (95% CI: [4.4%–6.8%]) of genes contained at least one exon pair with a Fisher's exact test *P*-value ≤5 × 10^−7^ and an absolute value log_2_-odds-ratio ≥ 0.5. This percentage rose gradually to 41.4% (95% CI: [23.5%–59.3%]) for exon pairs with ≥1000 reads (Fig. 7A). Genes can contain multiple such exon pairs, which introduces data structure. Using only one exon pair per gene (Methods) and repeating the estimation process, we recovered the same trend, albeit with lower percentages, because the retained exon pair is not necessarily a coordinated one (Fig. 7B). These trends (Fig. 7A,B) suggest two nonmutually exclusive hypotheses: Highly expressed genes could contain more coordinated exon pairs than lowly expressed genes—or given ≥1000 reads per gene, we would observe 23.5%–59.3% of tested genes with coordination. To distinguish between these scenarios, we performed down-sampling experiments (Methods), in which we sampled ≥25 reads from exon pairs with ≥500 reads. This resulted in a down-sampled list of exon pairs with reads drawn from the “≥500 informative reads” exon pair list that had an identical distribution of total informative reads as the original “≥25 reads” exon pair list. We repeated this 50 times and recorded the fraction of genes with coordination in each repetition. These down-sampled exon pairs (of ≥25 reads) behaved similarly to the real exon pairs of ≥25 reads and dissimilarly to the real exon pairs of ≥500 reads (Fig. 7C). This shows that, with ≥1000 reads per exon pair, we expect to observe 23.5%–59.3% of tested genes with coordination.

## Discussion

Isoform sequencing is influenced by five parameters: throughput, sequence quality, read-length, faithful quantification, and input requirements. We have used PacBio (Sharon et al. 2013; Tilgner et al. 2014) and SLR-RNA-seq (Tilgner et al. 2015), and Oxford Nanopore has recently been employed (Oikonomopoulos et al. 2016). SLR-RNA-seq provided 5 million ∼1.9-kb average reads and statistics on a gene-by-gene basis. SpISO-seq provides an almost full-length description of 17–25 million molecules (estimated from 11.6–16.7 million spliced molecules) and can be scaled to 50–100 million molecules with more input cDNA. Sequence quality is determined by Illumina sequencing and could be raised by combining multiple overlapping short reads into an assembly. Full-length PCR is not employed but only linear amplification, which initiates at multiple positions on the molecule. Each amplification event provides useful data, even when it fails to extend the entire molecule. This leads to high correlation of spISO-seq and short-read expression and is likely to limit bias against longer molecules, due to the necessity to complete full-length PCR cycles in the SLR-RNA-seq approach (Tilgner et al. 2015) or due to preferential sequencing of smaller molecules with PacBio (Eid et al. 2009). In summary, with more assessed molecules, spISO-seq has advantages for statistics and biological discovery. However, with sparser sequencing of all molecules, a full-length single sequence of the molecule is not (yet) provided—rather mapping is performed using unassembled short reads, which may be problematic for pseudogenes and repetitive regions.

Input requirements for spISO-seq (and SLR-RNA-seq) of 100 pg–1 ng of cDNA make prior amplification unnecessary in most situations and limit bias. SpISO-seq reveals more molecules of important gene classes such as lincRNAs and antisense genes (Mercer et al. 2009)—but for pseudogenes, mappings are error-prone, and long SLRs (Tilgner et al. 2015) or PacBio reads (Eid et al. 2009; Au et al. 2012; Koren et al. 2012; Sharon et al. 2013) offer advantages.

We confirm SLR-RNA-seq-based splicing coordination observations (Tilgner et al. 2015), although some disagreement exists: (1) complex splicing events (where, in addition to exon inclusion and exclusion, we observe intron retention or alternative splice sites), and (2) when a splicing event cannot be mapped by short reads. For the AD-associated gene *BIN1*, we confirm coordinated splicing (Tilgner et al. 2015), and we can now appreciate coordination in *MAPT*—a gene central to tauopathies (Rademakers et al. 2004; Wang and Mandelkow 2016).

Using the GENCODE version 24 annotation, we have found that ∼60% of all protein-coding genes contain two (or more) alternative events of exon usage that are separated by constitutive exons. Here, we have focused on pure pairs of exon-skipping events and genes, for which our previously published SLRs indicate two pure alternative exon-skipping events and for which we have at least 25 molecules in our spISO-seq data. Thus, we test 4624 exon pairs in 1354 genes and find 322 exon pairs in 125 genes to be coordinated. This is certainly only the tip of the iceberg, as there are thousands of genes—for which we do not possess 25 or more full-length molecules—and because 25 molecules allow detection of only close-to-perfect coordination.

Outstanding questions concerned the consequences and extent of coordination: We observe coordination events mostly in coding regions—and to a lesser extent in UTRs. The reasons for this observation are unclear for the moment, but it is possible that NMD is responsible for ∼33% of the observed coordinated cases in coding regions by degrading transcripts with a frame shift (see above). NMD would affect exon pairs with noncoding sequence comparatively less and contribute to increased coordination in coding regions. Alternatively (and not mutually exclusively), a lower evolutionary pressure to maintain coordination can be envisioned for UTRs and lincRNA genes—which would mean that coordination is, above all, contributing to control protein-coding sequences. The observation of lower coordination in noncoding sequence is supported by analysis of first and last alternative splice sites. We tested the first alternative donor and the last alternative acceptor of each gene for coordination. Despite the design to find dependencies involving the first and last splice site, a majority of coordination cases involved only splice sites of internal exons. However, dependencies between a first or last splice site and an internal splice site were also observed. The first donor and the last acceptor may be linked to alternative poly(A)-sites and TSS choice (e.g., Fig. 6D). Thus, the functional association may in fact be between the TSS and internal exon choice as previously shown (Cramer et al. 1997; Fededa et al. 2005) or between the internal exon and poly(A)-site, as previously hypothesized (Tilgner et al. 2012). Such situations we had previously not dealt with because we feared bias due to considerable length differences (Tilgner et al. 2015). Interestingly and consistent with universal alternative splicing in lincRNAs (Deveson et al. 2017), we do not observe coordination in lincRNAs, but deeper isoform data may reveal lincRNA coordination also. With infinite sequencing depth, we would expect a larger number of genes to display splicing coordination, unless lowly expressed genes follow a different paradigm than highly expressed genes. Down-sampling experiments suggest, however, that lowly expressed genes behave similarly to down-sampled cases of highly expressed genes. Thus, with deeper sequencing we will find considerably larger numbers of coordinated events (23.5%–59.3% of tested genes with ≥1000 reads).

Multiple distinct models can underlie non-NMD-mediated coordination events. First, it is well established that the same splicing factor can cause exon inclusion or exclusion depending on whether it is binding upstream, downstream, or within the exon (Ule et al. 2006; Llorian et al. 2010). Thus, a splicing factor binding site located upstream of the first alternative exon and downstream from the second alternative exon would lead to a mutually exclusive and thus coordinated pattern of both alternative exons. Similarly, concordant positioning of splicing factor binding sites could cause both exons to be included in the same molecules. Second, splicing outcome at one of the exons may leave molecular cues that influence splicing of the second alternative exon. One instance of this has already been established in the fibronectin gene (Fededa et al. 2005), in which inclusion of the upstream exon influences the downstream exon and in which promoter and transcription elongation rate influence coordination. Other molecular communication types, such as RNA structures linking alternative exon pairs, could potentially also play a role. Third, since our observations are based on bulk tissue isoform profiling, it is possible that some isoform abundances are determined by the abundances of distinct cell types and that these abundances lead to the observation of coordination. Last but not least, co-expression patterns of the splicing factors controlling the two exons of an exon pair could also play a role in shaping isoforms and coordination.

## Methods

Note that all experiments described here have been performed on a 10x Genomics GemCode instrument, but we also describe in the Methods experiences with the Chromium instrument (see Supplemental Methods).

### cDNA generation

cDNA was generated in a similar fashion as previously reported. A detailed protocol is found in the Supplemental Methods.

### GemCode whole transcriptome library preparation and sequencing

Sample indexing and partitioning, thermal cycling, final library generation, and quantification were done by following the 10x Genomics GemCode platform protocol (10x Genomics). Briefly, GemCode Gel-Bead Strip, GemCode Reagent mix, Primer Release Agent, and Surrogate Fluid were brought to room temperature. Freshly prepared GemCode creation master mix containing nuclease-free water, GemCode Reagent Mix, Primer Release Agent, and GemCode Polymerase was added into five individual PCR 8-tubes. One microliter of the 1000-, 500-, 250-, 125-, and 62.5-pg/µL of the purified dscDNA samples was loaded into each PCR tube and mixed well by slow pipetting on ice, spun quickly, and placed back on ice. GemCode Gel-Bead Strips were vortexed at full speed for 25 sec and collected in the bottom of the tubes and checked carefully for bubbles. Sixty microliters of sample mix were loaded into the sample wells in the GemCode Chip, followed by loading 85 µL of the GemCode Gel-Beads suspension into the designated row in the GemCode Chip. At least 150 µL of the partitioning oil was also loaded into the GemCode Chip. The GemCode Chip was covered with the GemCode Chip Gasket and inserted into the GemCode Instrument for massively partitioning into molecular reactors to extend the DNA and introduce specific 14-bp partition barcodes. One hundred fifteen microliters of GEM reactions were transferred into a 96-well PCR plate and sealed using Bio-Rad PX1 Plate Sealer to 185°C for 6 sec. The sealed PCR plate containing the GEM reaction was placed into a Bio-Rad C1000 Touch Thermalcycler with a 96- deep well reaction module, which was kept at a 4°C hold stage and amplified by incubating at 95°C for 5 min; 18 cycles of 4°C for 30 sec, 45°C for 1 sec, 70°C for 20 sec, and 98°C for 30 sec; then held at 4°C and purified using DynaBeads MyOne SILANE and SPRIselect Reagent (0.6× volume) following the GemCode protocol, and final purified partitioned, indexed, and amplified libraries were eluted in 52.5 µL of Elution Solution II.

In the following steps of the library construction, the P7 adapter containing the sample index was added to the GemCode Libraries. Briefly, the purified GemCode Libraries were sheared to 500 bp (E220 Focused-ultrasonicators) using Intensity: 5; duty factor: 5.0%; cycles per burst: 200; treatment time; 35 (sec), temperature: 7.0°C; sample volume: 50 µL. The sheared libraries went through end repair, 3′ ends tail adenylation, universal adapter ligation, post-ligation 0.8× SPRI cleanup, sample indexing PCR, and post-sample index PCR 1× SPRI Cleanup according to the manufacturer's instruction, and were eluted in 30.5 µL of Elution Solution III. Final libraries were quantified by a Agilent High Sensitivity DNA kit (#5067-4626) and also KAPA Illumina Library Quantification Kit-Universal (#KK4824).

The final GemCode Illumina-ready sequencing libraries were run individually on seven lanes of the Illumina HiSeq 4000 with paired-end 2 × 98-bp, 14-bp I5, and 8-bp I7 reads.

### Mapping and primary spliced molecule number estimation of microfluidic molecules

Barcoded short reads were aligned to the genome using STAR (Dobin et al. 2013). Short reads carrying the same barcode and being aligned to the same gene were considered to have originated from the same molecule. Gene-barcode pairs for which conflicting spliced reads were found were labeled as “collisions” and removed from the analysis. The total number of molecules was estimated by counting the number of gene-barcode pairs, for which at least one (resp. 2,3,4,…100) spliced read(s) was available for the gene (resp. barcode). Further details are available in the Supplemental Methods.

### Calculation of molecules per million

For each spliced gene, we counted the barcodes that identified this gene (as defined by a splice read identifying a GENCODE intron of this gene) and divided this count by the total number of detected molecules, in millions.

### Length estimations of gene classes

Based on the GENCODE version 24 annotation (5, 6), we chose for each gene's length the length of its longest transcript (excluding introns).

### High-confidence identification of splicing events in individual molecules

We mapped the linked short reads to the GRCh38 version of the human genome and annotated GENCODE v24 spliced junctions, requiring at least mapped 6 bp on either side of the junction to decrease possible false-positive mappings.

### Coordination of alternative internal exons

For each exon pair, we determined the number of barcodes that (1) included both exons, (2) included the first but not the second exon, (3) included the second but not the first exon, and (4) skipped both exons. These numbers were used to construct a 2×2 table. A Fisher's exact test was performed, and correction for multiple testing was performed with the Benjamini-Yekutieli method (Benjamini and Yekutieli 2001). For the same exon pairs, we constructed a 2×2 table, followed by statistical testing and correction for multiple testing using spISO-seq data. More detail is available in the Supplemental Methods.

### Coordination between first alternative donors and last alternative acceptors

For each gene we determined all alternative acceptors and all donors within the gene. If the alternative donor was located upstream of the alternative acceptor and in all molecules there was at least one intermediate exon in between the two splice sites, we retained the splice site pair, thus focusing on events in which the two alternative splice sites are defined by distinct splicing reactions. For the same exon pairs, we constructed a 2×2 table, followed by statistical testing and correction for multiple testing using spISO-seq data. More detail is available in the Supplemental Methods.

### Down-sampling experiments

Each exon pair is represented by a 2×2 table with the numbers *a* and *d* on the descending diagonal and *b* and *c* on the ascending diagonal; *a* is defined as the number of reads including both exons, *d* as the number of reads skipping both exons, *b* as the number of reads including only exon 1 (and skipping exons 2), and *c* as the number of reads including only exon 2 (and skipping exon 1).

For each 2×2 table *t*, we defined *n*_*t*_ = *a*_*t*_ + *b*_*t*_ + *c*_*t*_ + *d*_*t*_ as the informative reads for this table *t*. In order to avoid biases caused by genes with many significant tables (like *BIN1*), here, for each gene we only use the table (or exon pairs) for which *n*_*t*_ is highest among all tables (or exon pairs) for the gene in question.

From the real data, we defined two lists of tables: *L* (for low) being the list of tables *t* for which *n*_*t*_ ≥ 25, and *H* being the list of tables *t* for which *n*_*t*_ ≥ 500. Trivially, each element of *L* is also an element of *H*. For *H* and for *L* separately, we computed the percentage of genes that have a *P*-value of ≤5 × 10^−7^ and abs[log_2_((*a*+0.5) × (*d*+0.5)/(((*b*+0.5) × (*c*+0.5)))] ≥ 0.5. We use a pseudocount of 0.5 to be able to calculate the log-odds-ratio even when a zero appears in the table. These two percentages are shown as arrows in Figure 6C. The cutoff of 5 × 10^−7^ was chosen because, with this cutoff, one would be able to implement a Bonferroni correction for 100,000 tests. The number of 100,000 tests is a reasonable upper bound estimate on the number of alternative exon pairs (that are separated by constitutive exons), given 20,000 protein-coding genes.

We then defined a down-sampled list as follows:

For each table *l* in *L*, we chose one table randomly among the tables *h* in *H* that verified *n*_*h*_ ≥ *n*_*l*_. From this table we chose *n*_*l*_ reads randomly and thus obtained a table *ds* that has *n*_*l*_ total counts and that is down-sampled from *h*. The list of down-sampled tables *ds* defines a list of tables (*DS*) that has the identical distribution of informative reads as *L* (but, by construction, not necessarily similar distributions of the *a, b, c*, and *d* values). For this list *DS*, we computed the percentage of genes that have a *P*-value of 5 × 10^−7^ or less and abs[log_2_((*a*+0.5) × (*d*+0.5)/(((*b*+0.5) × (*c*+0.5)))] ≥ 0.5.

We repeated this process 50 times to define 50 representations of down-sampled lists and their associated percentages. This distribution is shown in Figure 6C in blue.

## Data access

The sequence data from this study have been submitted to the NCBI BioProject database (http://www.ncbi.nlm.nih.gov/bioproject/) under accession number PRJNA267017 (SRA accession numbers SRX3207157–SRX3207163).

## Competing interest statement

M.S. is a cofounder of Personalis, SensOmics, and Qbio, and an advisory board member of Personalis, SensOmics, Qbio, and Genapsys. M.R. is an employee of ArcBio.

## Supplementary Material

Supplemental Material
