# Supplementary material for: Microfluidic isoform sequencing shows widespread splicing coordination in the human transcriptome
Source: Genome Res. 2018 Feb;28(2):231–42. doi: 10.1101/gr.230516.117 (PMC5793787; doi:10.1101/gr.230516.117)
Supplement: Supplemental Material [file supp_28_2_231__index.html]

Microfluidic isoform sequencing shows widespread splicing coordination in the human transcriptome — Supplemental Material 

# Microfluidic isoform sequencing shows widespread splicing coordination in the human transcriptome

## Supplemental Material

- Supplemental\_Methods\_and\_Figures.pdf
- Supplemental\_Table\_S2.zip
- Supplemental\_Table\_S1.zip
- Supplemental\_Table\_S3.zip
