# Supplementary material for: Microfluidic isoform sequencing shows widespread splicing coordination in the human transcriptome
Source: Genome Res. 2018 Feb;28(2):231–42. doi: 10.1101/gr.230516.117 (PMC5793787; doi:10.1101/gr.230516.117)
Supplement: Supplemental Material [file supp_gr.230516.117_Supplemental_Methods_and_Figures.pdf]

Supplemental Materials
For
Microfluidic isoform sequencing shows widespread splicing coordination in the human transcriptome.

Hagen Tilgner[1*+], Fereshteh Jahanbani[2*], Ishaan Gupta[1], Paul Collier[1], Eric Wei[2], Morten Rasmussen[3], Michael Snyder[2+]

I. Supplementary figures 2
Supplemental figure S1 2
Supplemental figure S2 3
Supplemental figure S3 4
Supplemental figure S4 5
Supplemental figure S5 6
Supplemental figure S6 7
II. Methods 8
Experimental methods on the GemCode system 8
1st and 2nd strand cDNA synthesis 8
Experimental recommendations for the Chromium system 9
1st and 2nd strand cDNA synthesis 9
Bioinformatics analysis 10
Re-mapping of previously published long read 10
Primary spliced molecule number estimation of microfluidic molecules 11
Coordination of alternative internal exons using SLR-RNA-seq. 11
Coordination of alternative internal exons using spISO-seq 12
Distribution of purely coding exon pairs and pairs involving non-coding sequence matched for informative molecules 13
Coordination between first alternative donors and last alternative acceptors 13
Supplementary References 14

I. Supplementary figures

Supplemental figure S1

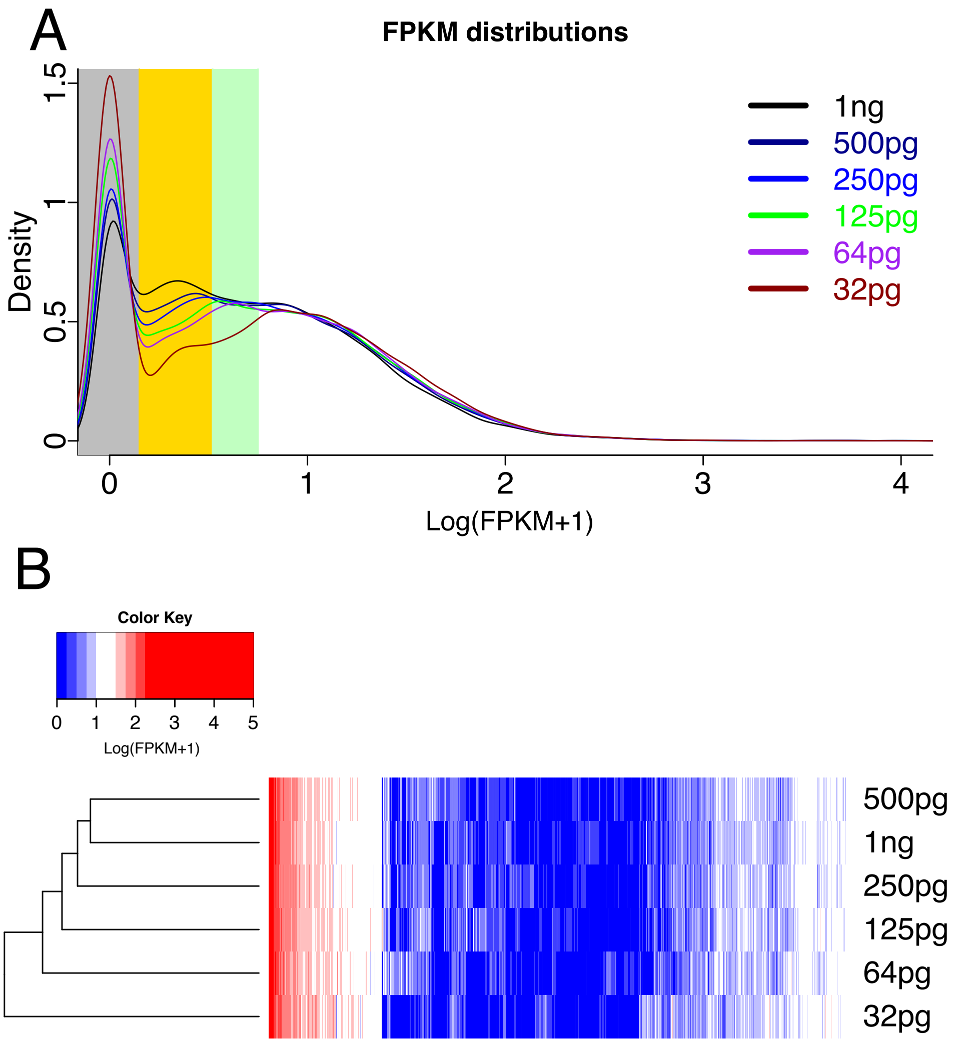

Figure S1: MiSeq exploration of GemCode behavior with low inputs. (A) Density plots of FPKMs with six input amounts to the system. **(B)** Heatmap of gene expression values (FPKM) across all six samples.

Supplemental figure S2

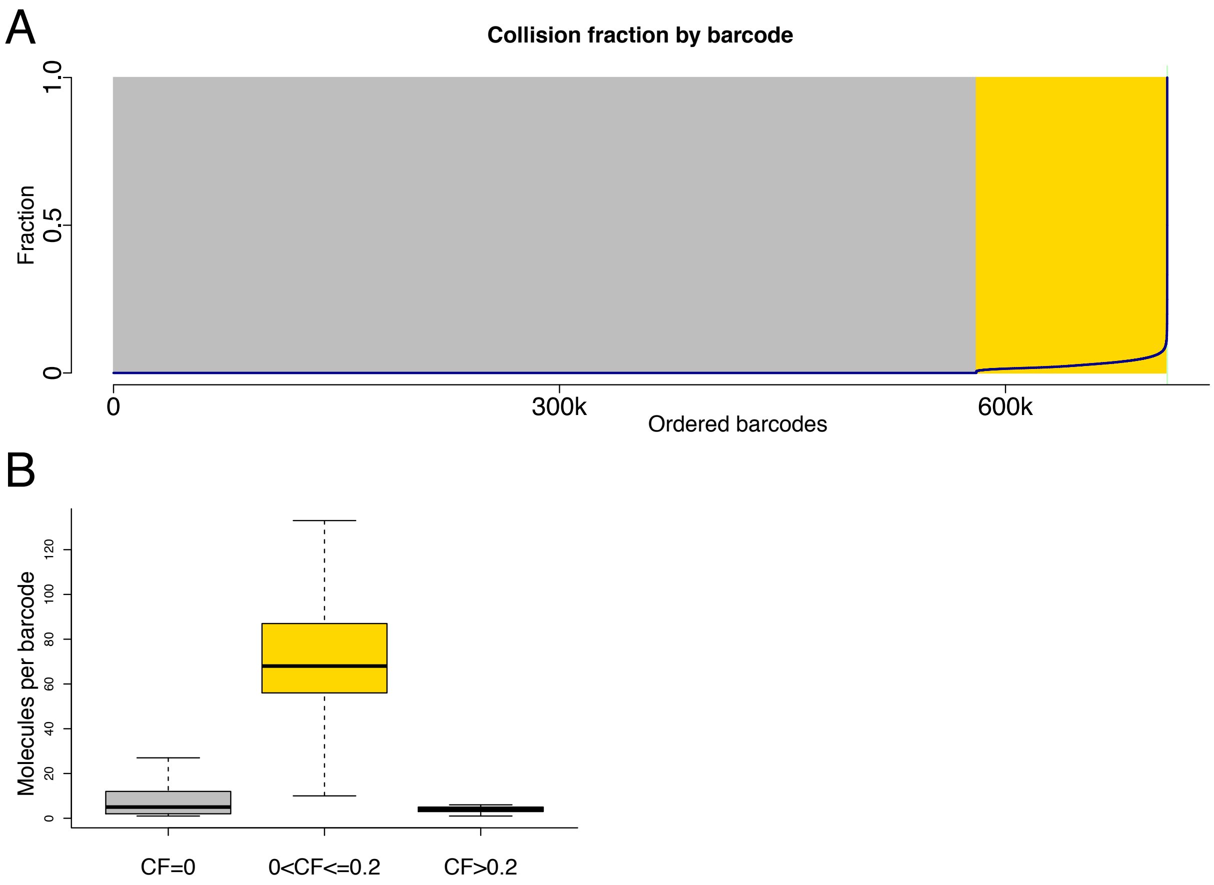

Figure S2: Collision fraction by barcode. (A) Fraction of genes for each barcode that showed a collision. Barcodes are ordered by collision fraction. Gray area is enriched in false positive barcodes. **(B)** Number of genes for many barcodes without collisions, many barcodes with few collisions and for very few barcodes with many collisions.

Supplemental figure S3

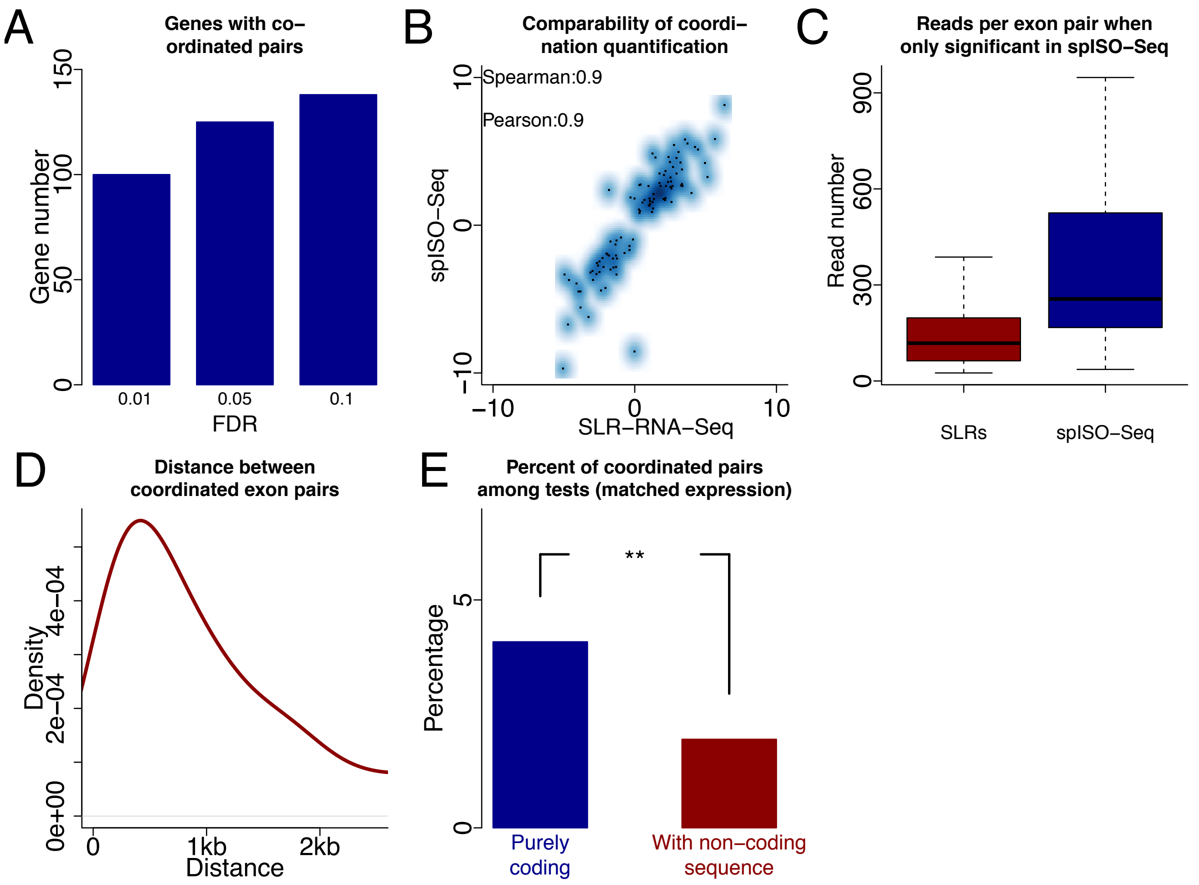

Figure S3: Coordination of exon pairs. (A) Number of genes with coordination events in the spISO-seq data at different FDR values. **(B)** Dotplot for extent of coordination according to SLR-RNA-seq and spISO-seq for cases, in which only spISO-seq indicates coordination. **(C)** Number of informative molecules (left: SLR-RNA-seq, right: spISO-seq) for genes with coordination only revealed by spISO-seq. **(D)** Distribution of number of bases on mature, annotated GENCODE transcripts that lie between the two alternative exons (averaged over all GENCODE transcripts that start before the upstream exon and end after the downstream alternative exon**. (E)** Percent of purely protein-coding exon pairs that are coordinated and of exon-pairs that contain non-coding sequence. In this analysis, we chose exon pairs in both distributions so that the underlying distributions of informative reads in the two categories are identical.

Supplemental figure S4

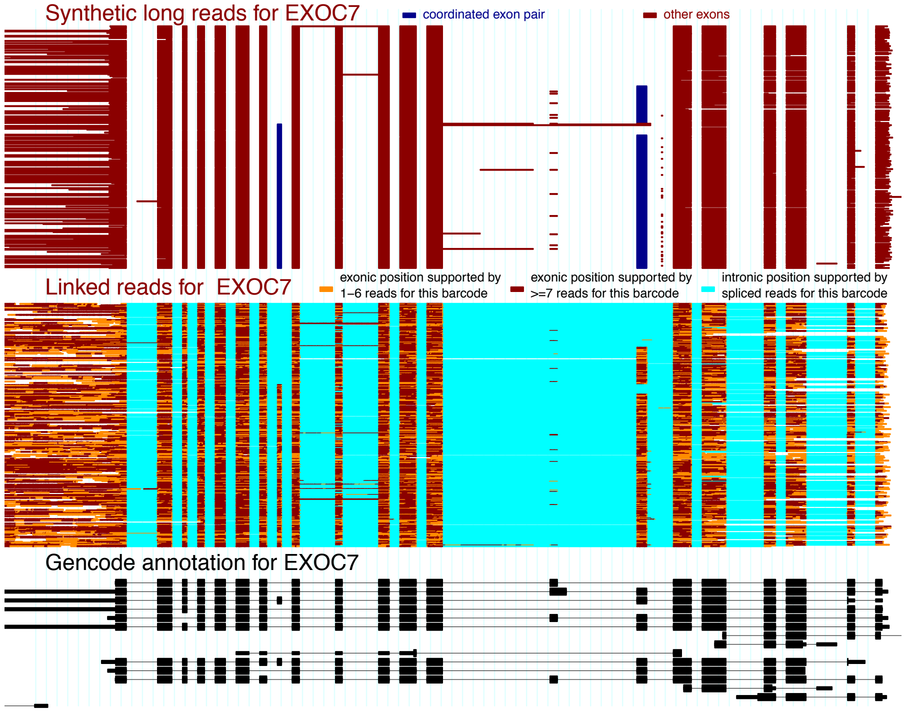

Figure S4: EXOC7 example of coordination. Single gene view for the EXOC7 gene, Bottom, black track: GENCODE annotation. Middle, colored track: spiSO-seq data, with each line representing one molecule. Top, red-brown track: SLR-RNA-seq data with each line representing one molecule.

Supplemental figure S5

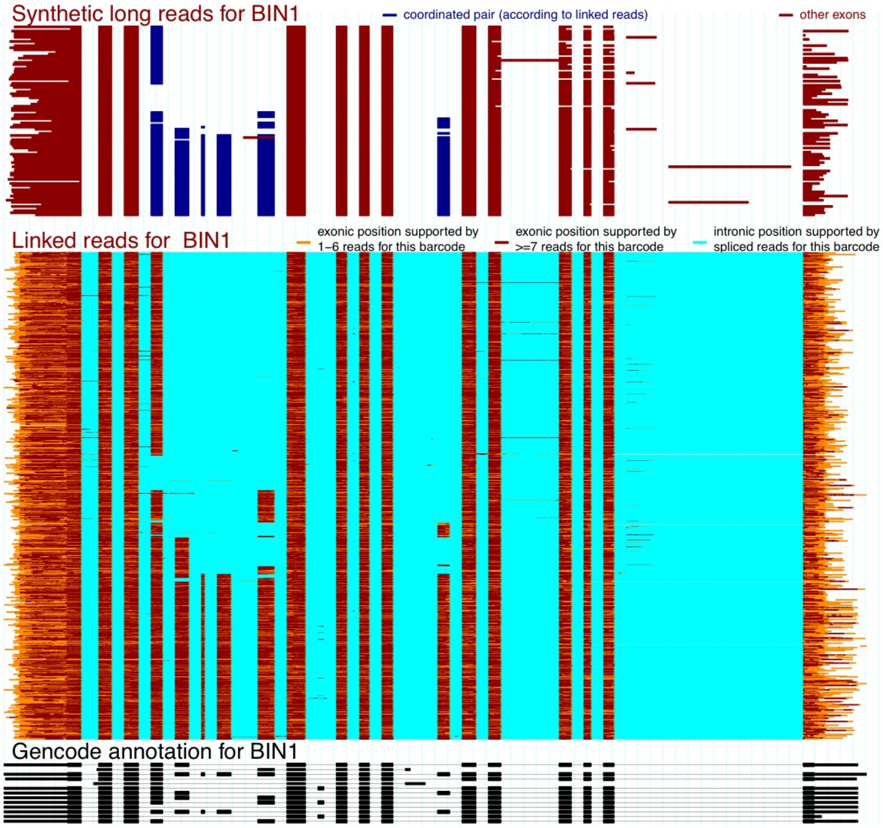

Figure S5: BIN1 example of coordination. Single gene view for the BIN1 gene, Bottom, black track: GENCODE annotation. Middle, colored track: spiSO-seq data, with each line representing one molecule. Top, red-brown track: SLR-RNA-seq data with each line representing one molecule.

Supplemental figure S6

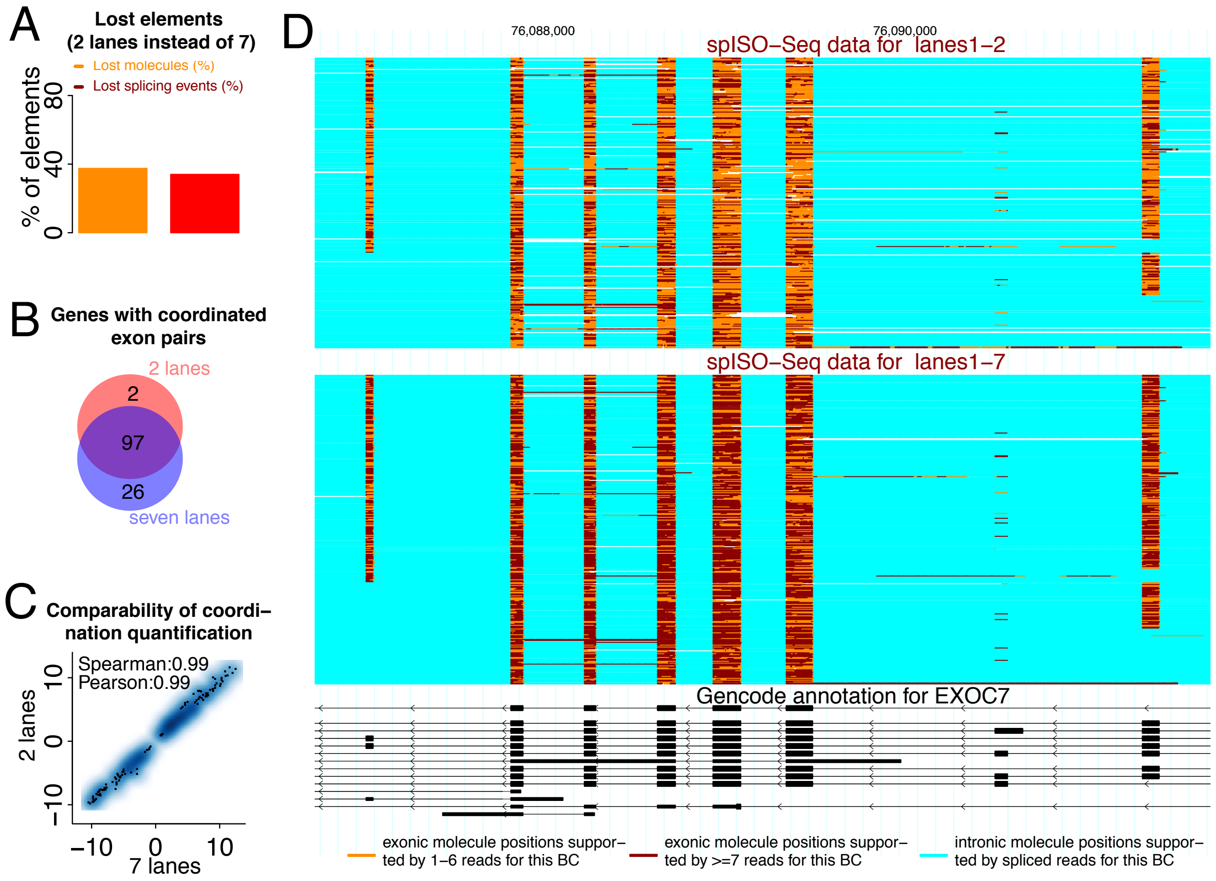

Figure S6: Effects of devoting less sequencing power to the same number of molecules. (A) Molecules and splicing events lost when using only 2 Illumina lanes instead of seven **(B)** Overlap of coordinated genes when using seven and two lanes for sequencing the same molecules. **(C)** Dotplot for extent of coordination when using all 7 and only two genes for genes with coordination using seven and using two lanes. **(D)** Single gene view for the EXOC7 gene, Bottom, black track: GENCODE annotation. Middle, colored track: spiSO-seq data using all 7 lanes, with each line representing one molecule. Top colored track: spiSO-seq data using only 2 lanes, with each line representing one molecule.

II. Methods

Experimental methods on the GemCode system

1st and 2nd strand cDNA synthesis

mRNA was isolated from DNase-treated human brain total RNA (Ambion First Choice Human Brain Reference RNA, Cat No: AM6050) using FastTrack MAG mRNA Isolation kit (Life Technologies, Cat No: K1580-01). The purified mRNA integrity was assessed with the Agilent RNA 6000 Nano Assay kit on the Agilent 2100 Bioanalyzer (Agilent Technologies, Cat No: 50674626). The purified mRNA was converted into sscDNA using Clontech SMARTer™ PCR cDNA Synthesis Kit (Cat. Nos. 634925) following the manufacture protocol. Briefly, 100 ng of purified mRNA was incubated with 1 µl 3' SMART CDS Primer II A at 72 °C for 3 min and transferred immediately to ice. The cDNA synthesis master mix, which contained Clontech SMARTScribe reverse transcriptase, 5X First-Strand Buffer, DTT, dNTPs and SMARTer II A Oligonucleotide, was then added to the mixed of RNA and the 3' SMART CDS Primer II A, and incubated at 42 °C for 90 min. The reaction was terminated by incubating at 70 °C for 10 min. The entire full length sscDNA library was converted into dscDNA products by adding the PCR master mix containing 10X Advantage 2 PCR Buffer, dNTP mix, 5' PCR Primer II A (12 µM) and Advantage 2 Polymerase mix and incubating at 95°C for 1 min, 95°C for 15 sec, 65°C for 30 sec and 68°C for 3 min. dscDNA library was purified with 0.8X volume of Agencourt AMPure XP beads (Beckman Coulter, Cat No. A63881). The quality of the dscDNA was measured and validated by the Agilent DNA high

sensitivity kit on the Agilent 2100 Bioanalyzer (Agilent Technologies, (Cat. No. 5067-4626) and used as in out for spISO-seq library generation on 10x Genomics instrument.

Experimental recommendations for the Chromium system

Please note that all experiments that were analyzed for biological results were obtained on the 10x Genomics GemCode system. While we were analyzing this data 10x Genomics released the updated Chromium system (version 2 being the current up-to-date version). In house, we noticed different behavior, which we are detailing here. Please also note, that some of the experimental procedure was changed for cost efficiency. In principle the changed steps are equivalent to the above procedure.

1st and 2nd strand cDNA synthesis

Total RNA was extracted by use of Trizol LS (Invitrogen Cat#10296028) and chloroform to obtain phase separation into aqueous phase. RNA extraction from aqueous phase was performed with RNA Clean and Concentrator (Zymo Cat#R1015) following manufactures protocol. Total RNA was quantified by Qubit (Invitrogen Cat#Q32852) for quantification and run on the Fragment Analyzer (AATI Cat#DNF-472) for quality assessment then diluted to input concentrations of 40ng/ul. To synthesize first strand cDNA we used a total input RNA concentration of 100ng and followed the SmartSeq2(*1*) protocol with the following modifications. The Oligo(dT) primer was diluted to 2,5nM and 1ul used for the priming, for the RT 0,2ul of LNA TSO Oligo was used. Second strand synthesis was performed using Kapa HiFi HotStart Ready Mix (Kapa Biosystems Cat#KK2600) primed with ISPCR primers and used the following thermal conditions, 98oC for 3min then 6 cycles of 60 oC for 5min, 72 oC for 20min followed by a final extension of 72 oC for 30min and held

at 10 degrees. RNase H digestion was preformed after second strand synthesis to remove any RNA-DNA hybrid molecules by addition of 1U of RNase H (Thermo Scientific Cat#EN0201) and incubated for 30min at 37 oC. The final dscDNA library was quantified on Qubit and size distribution was assessed using the Fragment Analyzer and diluted in 10ul to 1ng/ul, 500pg/ul, 250pg/ul and 125pg/ul respectively for input into the 10x Genomics Genome protocol. We recorded the number of uniquely mapping read pairs and compared them to the ones obtained from the GemCode system. Note, that here we use very stringent mapping parameters to determine exact intron positions, not the more relaxed ones used in Figure 1 for molecule identification.

Sample Concentration	Uniquely mapped read pairs (Chromium)	Uniquely mapped read pairs (GemCode)
1 ng/ul;	52.52%	
0.5 ng/ul	29.89%	
0.25 ng/ul	23.05%	
0.125 ng/ul	18.11%	57.04%-58.79% (across seven lanes)

As of now, we therefore recommend to use 1ng/uL when using the Chromium instrument (genome v2 chemistry) with 8 lanes of sequencing.

Bioinformatics analysis

Re-mapping of previously published long read

Re-mapping of SLR-RNA-seq(*2*) data was performed using GMAP(*3*) as described previously(*2*).

Primary spliced molecule number estimation of microfluidic molecules

To estimate the total number of spliced RNA molecules, we mapped the linked short reads to the GRCh38 version of the human genome and annotated GENCODE v24 spliced junctions using STAR(*4*). For this primary molecule number identification step (but not for subsequent coordination analysis steps), we used rather permissive parameters, considering a junction identified when it was covered by a spliced short read with at least 1nt on both sides of the intron. A spliced molecule was considered identified when at least one of its gene's intron was observed in this way for the barcode in question. Obviously a wrongly assigned short read, as for example a fragment originating from the parent gene being assigned to the pseudogene can lead to a false positive identification.

Coordination of alternative internal exons using SLR-RNA-seq.

We first considered all exon pairs that appeared at least once as an internal exon of a synthetic long read. We then calculated a PSI value for both splice sites of each exon as well as the entire exon and retained only exons, for which

- all PSI values were between five and 95%.
- The exon inclusion and the exon exclusion isoform represented 80% of all overlapping molecules – thus discarding exons with frequent intron retention or alternative acceptors or donors

We then considered non-overlapping exon pairs coming from the same gene and counted read numbers (that had an intermediate exon), which

- Included both exons (as judged by usage of the donor of the first exon and the acceptor of the second exon)
- Included the first exon and skipped the second exon
- Skipped the first exon and included the second exon

- Skipped both exon

For exon pairs that had at least 25 such reads we performed a two-sided fisher test and corrected for multiple testing using the Benjamini-Yekutieli correction(*7*).

Coordination of alternative internal exons using spISO-seq.

All exon pairs, for which we recorded informative read numbers (see section "Coordination of alternative internal exons using SLR-RNA-seq") and for which we had at least 5 overlapping informative reads were considered for spISO-seq analysis, based on the thought that the increased sequencing depth of spISO-seq would allow the determination of significant events even when low SLR counts were observed.

Similarly to the above procedure, we counted linked reads, which

- Included both exons (but this time as judged by observation of at least the acceptor or the donor of each exon)
- Included the first exon and skipped the second exon
- Skipped the first exon and included the second exon
- Skipped both exon

Note, that this counting procedure is different from the one employed for SLR-RNA-seq. For SLR-RNA-seq (as would be for Pacific Biosciences or Oxford nanopore), if a long read is informative about the usage of the donor of the downstream exon, it is necessarily informative about the acceptor of that same exon. For SLR-RNA-seq we thus focused on the acceptor. For spISO-seq, due to the sparser coverage of the molecule, we may encounter situations, where we can tell if the donor of the downstream exon is used but due to random chance no linked short read is available informing about the acceptor. We thus chose to consider the exon as present if the acceptor or the donor were observed. This can introduce

discrepancies between the SLR-RNA-seq and the spISO-seq approach. It is possible to use identical counting procedures, but only by limiting the power of the spISO-seq approach.

Distribution of purely coding exon pairs and pairs involving non-coding sequence matched for informative molecules

We generated two lists of tested exon pairs: (i) Those that contained non-coding sequence and (ii) those that only contained coding sequence according to the GENCODE v24 annotation(*5*, *6*) and counted for each list the number of informative linked reads. We randomly picked one exon pair of the first list and one of the second list that had the same number of informative reads. We repeated this procedure until no more pairs of exon pairs were available that had identical informative read numbers. The chosen pairs of exon pairs now defined two lists of exon pairs (one with non-coding sequence, the other without) that had identical distributions of informative reads.

For each list we calculated the distribution of p-values (with each p-value being a measurement of the extent of coordination) and subjected the two distributions to a two-sided Wilcoxon rank sum test.

Coordination between first alternative donors and last alternative acceptors

We considered for each gene the first annotated donor and the last annotated acceptor that was alternative in our SLR-RNA-seq data, where "being alternative" is defined as "at least 5% and at most 95% of all overlapping spliced molecules use the splice site in question". We retained only splice site pairs, for which all reads that overlapped both splice sites also had an intermediate exon with respect to the two splice sites.

Finally, we counted linked reads that

- used both splice sites
- used the first site and skipped the second

- did not use the first site and used the second
- did not use either site

The resulting 2x2 tables were subjected to a fisher test and corrected for multiple testing using the Benjamini Hochberg method(*8*). Note that in this procedure and contrarily to our SLR-RNA-seq procedure above a molecule that does not use a splice site because of intron retention is counted. This may lead to biases, because intron retention can drastically change the length of molecules. However the spISO-seq approach we present here should limit those problems, because no full-length amplification or even sequencing is needed.

Barcodes supporting introns from GENCODE version 24 and from reference(*2*) are available in Table S1. Note, that a large number of SLR-RNA-seq defined introns did not show barcode support, presumably due to differences in mapping strategies.

Supplementary References

1. S. Picelli *et al.*, *Nat. Protoc.* **9**, 171–81 (2014).
2. H. Tilgner *et al.*, *Nat. Biotechnol.* **33**, 736–42 (2015).
3. T. D. Wu, C. K. Watanabe, *Bioinformatics*. **21**, 1859–75 (2005).
4. A. Dobin *et al.*, *Bioinformatics*. **29**, 15–21 (2013).
5. J. Harrow *et al.*, *Genome Biol.*, in press, doi:10.1186/gb-2006-7-s1-s4.
6. J. Harrow *et al.*, *Genome Res.* **22**, 1760–74 (2012).
7. Y. Benjamini, D. Yekutieli, *Ann. Stat.* **29**, 1165–1188 (2001).
8. Y. Benjamini, Y. Hochberg, *J. R. Stat. Soc. Ser. B ...*, 289–300 (1995).
